# Evaluation of the Utility of the Random Amplified Polymorphic DNA Method and of the Semi-Specific PCR to Assess the Genetic Diversification of the *Gerbera jamesonii* Bolus Line

**DOI:** 10.1100/2012/450920

**Published:** 2012-04-19

**Authors:** Zbigniew Rusinowski, Olga Domeradzka

**Affiliations:** Department of Ornamental Plants, Warsaw School of Life Sciences, Faculty of Horticulture and Landscape Architecture, Warsaw University of Life Sciences, Nowoursynowska 159, 02776 Warsaw, Poland

## Abstract

An attempt was made to evaluate the utility of a method which employs semi-specific PCR using partially specific primers for the coding sequence (ET) at the exon-intron contact and of the RAPD method to identify eight Polish cultivars of gerbera. It was demonstrated that the PCR method which employs semi-specific primers is as simple and economical as the RAPD method, simultaneously the images of the multiplied by means of the semi-specific PCR method DNA fragments are more complex and polymorphic than those obtained through the RAPD method. The studies of the genetic diversification of *Gerbera* cultivars employing the aforementioned methods made it possible to conduct a concentration analysis and evaluation of the genetic distance between the lines, manifesting at the same time the superiority of the semi-random PCR method. Moreover, it transpired that the use of mixtures of RAPD primers not always leads to an increase of the number of generated polymorphic bands.

## 1. Introduction

At the turn of the 21st century, identification of cultivars and protection of breeders' rights became an important issue, especially when it is impossible to tell them apart by their morphologic features such as, for example, the shape of seeds, bulbs, or cuttings [1]. It frequently occurs that cultivars, phenotypically similar, for example, whose flowers are of the same color stem from different breeding companies, and may be confused. Thanks to modern methods based on DNA analysis techniques allow to identify and select plants bearing desired features [2]. The current methods quickly enable us to profoundly analyze of consanguinity and pedigree and to determine the value of breeding material [3]. RAPD is one of the methods recommended to identify the cultivars of *Gerbera jamesonii* [4, 5].

*Gerbera *has the current methods quickly allows for analysis of an important position in the floral market for several years. Each year new cultivars are introduced; they are to attract customers not only with their color but also with their shape and the size of their inflorescence and to attract growers with higher yield and tolerance to growing conditions. The preferences of Polish customers as for cut flowers have changed in recent years, and, after a short-termed stagnation which affected the gerbera market, we may witness a slow but significant rise interest among those who purchase these species.

 The present paper attempts to compare the usability of the RAPD method and semi-specific PCR with the use of partially specific primers for the coding sequence lying on the exon-intron junction, never hitherto applied in the case of the gerbera cultivar to identify eight Polish cultivars bred by the Pętoś company.

## 2. Materials and Methods

The experiment was conducted in the years 2004–2006 at the Faculty of Horticulture and Landscape Architecture at the Warsaw University of Life Sciences (*Wydział Ogrodnictwa* i *Architektury Krajobrazu SGGW*) in Warsaw. The material was delivered by the *Pętoś *“*Specjalistyczny Zakład Ogrodniczy Bartoszyce*” company. Eight new Polish plant cultivars raised by the aforementioned company were used (*Amelia, Bartoszyce, Bolesławiec, Delfin, Kraków, Kreta, Safona, Samuraj*).

 To isolate the DNA young leaves were used as source. Two DNA isolation methods were employed. The isolation by means of Genomic Mini AX plant kits from the A&A Biotechnology company and the cTAB procedure after Murray and Thompson [6].

 In the RAPD experiments, the amplification program consisted of 40 m cycles; initial denaturation 72°-5′, denaturation 94°-1′, attachment 39°-1′, final temperature 72°-2′ [7]. The composition of the amplification was performed in two-stages mixture PCR whose total volume equaled 25 *μ*L 15.95 *μ*L of water dd (miliQ), 2 *μ*L of MgCl^2^, 2.5 *μ*L of buffer 10x, 1.25 *μ*L of dNTP, 1 *μ*L of primer, 0.3 *μ*L of TAQ polymerase DNA, and 2 *μ*L of tested DNA (approx. 10 ng/*μ*L).

 In the case of semi-specific primers, the DNA amplification was two stage. At the first stage for the 15 nucleotide primers in comparison to the 18 nucleotide primers, the primer attachment temperature was by 10° higher (50–60°) at the second stage it was by 10° lower (64–54°) the attachment time was 1 minute. In both cases this amplification program was used: denaturation 94°-40′′, lengthening of the DNA chains in 72°-2′, multiplication completed in 72°-5′, at the second stage next denaturation followed in 94°-40′′, lengthening of the DNA chains in 72°-2′, and final multiplication 72°-10 [8]. The composition of the mixture of the semispecific PCR by Rafalski [8], of which volume requaled 20 *μ*L, 10 *μ*L of water dd (miliQ), 2 *μ*L of MgCl_2_, 2 *μ*L of buffer 10x with ammonium sulfate, 0.5 *μ*L of dNTP, 3 *μ*L of primer, 1 TAO unit of DNA polymerase, and 2 *μ*L of the tested DNA (approx. 10 ng/*μ*L). Reagents from MBI Fermentas were used, with the exception of dNTP bought from Invitrogen. The electrophoretic separation was conducted on 1.5% agarose gel in TAE 1x buffer with addition of 10 *μ*L 0.01% of ethidium bromide. The visualization took place in the UV light. The experiment was documented with the help of a monochromatic camera and software by Biometra BioDell.

 The genetic similarity of the analyzed cultivars was computed by means of the Nei and Li formula [9] of the mean UPGMA connections. The concentration analysis was performed with the use of the NTSYS-pc program, v. 2.1.

## 3. Results

### 3.1. The Diversification Characteristics of Eight *G. jamesonii* Cultivars by Means of Selected RAPD Primers

The usability of twenty 10-nucleotide RAPD primers prepared according to the operon nomenclature in the DNA Sequence-forming Laboratory at the Institute of Biochemistry and Biophysics *PAN *was tested. 

 The RAPD technique was used to differentiate eight gerbera cultivars. For the purpose of the study, 6 out of the 20 tested primers were chosen: those which generated the highest total number of bands and the highest number of differentiating bands (Table 1).

 The primer which gave the highest percentage of differentiating bands (40%) generating the lowest number of total bands was the A2 primer. Primer C11, which generated the highest number of bands (10), gave relatively small, equals 20%, contribution of differentiating bands. The other primers rendered on average 28.9% polymorphic bands (Figure 3).

**Figure 1:**
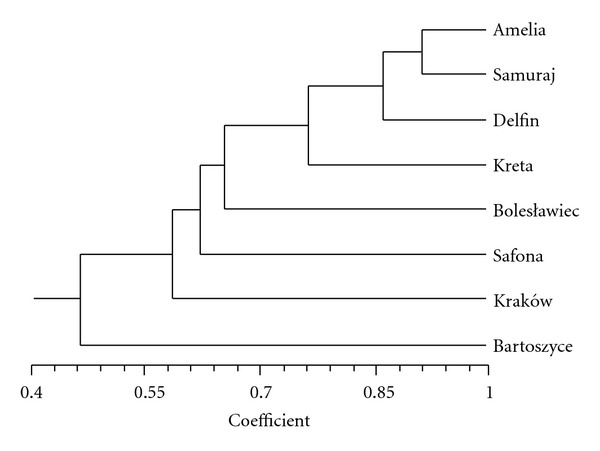
Similarity diagram of the tested *G. jamesonii *cultivars comuted from the mean connections method (UPGMA) and from a RAPD analysis of 19 DNA fragments.

The evaluation of genetic similarity of the cultivars was based on the analysis of 19 DNA fragments obtained from experiments using the RAPD method (Figure 1). The genetic distance coefficients between the tested lines were computed from the RAPD experiments and ranged from 0.09 to 0.62 (the average 0.37). It had been computed that the minimum genetic distance is between the *Amelia* and *Samuraj* cultivars (0.09), and the maximum distance separates the *Bolesławiec* from the *Bartoszyce *cultivar and the *Bartoszyce *from the *Safona *cultivar (0.62).

### 3.2. The Evaluation of the Differentiation of *Gerbera* sp. Cultivars by Means of Semi-Specific PCR

To distinguish the eight *G. jamesonii* cultivars, the utility of 12 semi-conservative primers was evaluated; those primers were partially specific for the coding fragment at the 15- and 18-nucleotide exon-intron junction (ET) designed by Dr. Andrzej Rafalski (Table 2) and prepared at the Institute of Biochemistry and Biophysics of the Polish Academy of Science (Instytut Biochemii i Biofizyki PAN).

 Primer ET 6/18 (Figure 4) yielded the highest total number of bands and included the majority of differentiating bands. Next was the ET 2/18 primer which generated 11 bands in all, 90.9% of which were differentiating. The other 18-nucleotide primers would generate from 4 to 9 bands of which 49.9% were polymorphic. In the 15-nucleotide group, primer ET 32/15 (Figure 5) yielded the highest total number of bands (10). Except for ET 31/15 (0), the remaining primers generated from 3 to 9 bands with the average of 35.1% of polymorphic bands.

**Figure 2:**
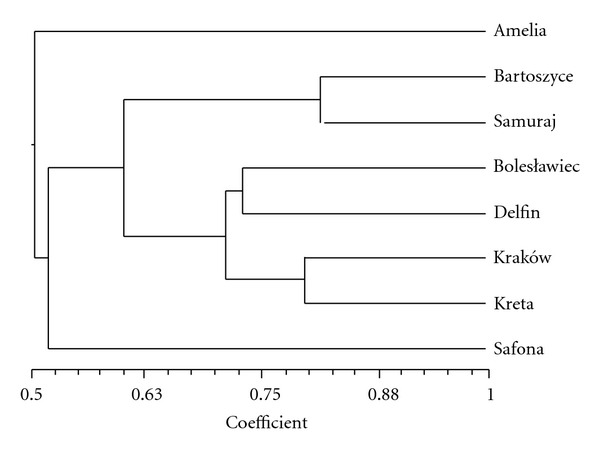
Similarity diagram of the *G. jamesonii* cultivars computed from the mean connections method (UPGMA) and from an analysis of 59 DNA fragments obtained by means of semi-specific PCR.

**Figure 3:**
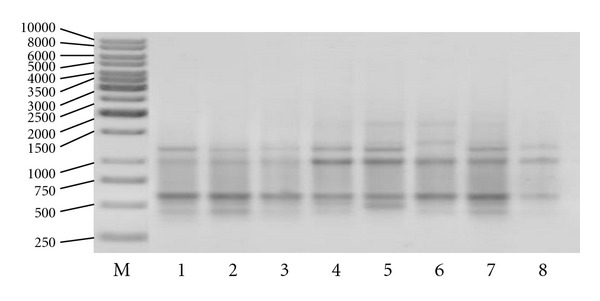
The amplification profile generated with the use of D11, from the left: marker (M) of the size 1 kb, *G. jamesonii* cultivars: 1 *Amelia, *2* Bartoszyce, *3 *Bolesławiec, *4* Delfin, *5 *Kraków, *6* Kreta, *7* Safona, and *8 *Samuraj*.

**Figure 4:**
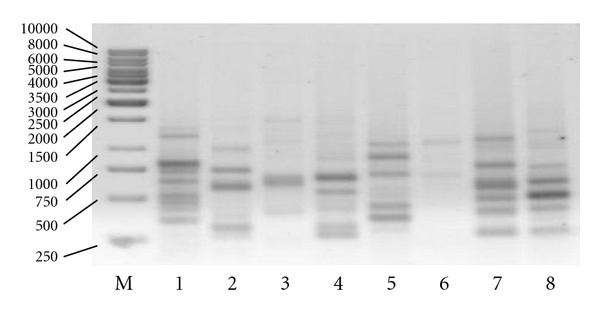
Amplification profile generated with the use of the primers ET 6/18, from the left: marker (M) of the size 1 kb, *G. jamesonii* cultivars: 1 *Amelia, *2* Bartoszyce, *3 *Bolesławiec,* 4* Delfin, *5 *Kraków, *6* Kreta, *7* Safona, and *8 *Samuraj*.

**Figure 5:**
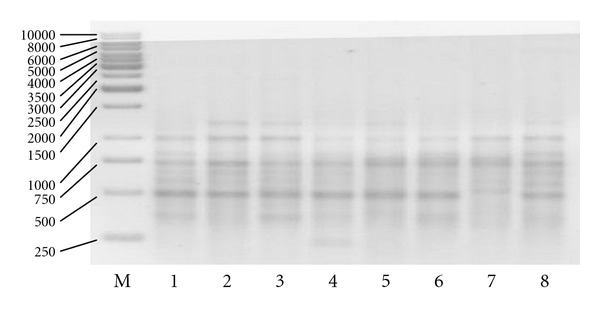
Amplification profile generated with the use of the primers ET 32/15, from the left: marker (M) of the size 1 kb,* G. jamesonii *cultivars: 1* Amelia, *2* Bartoszyce, *3* Bolesławiec, *4* Delfin, *5* Kraków, *6* Kreta, *7* Safona, and *8 *Samuraj*.

The evaluation of genetic similarity of the cultivars was based on the analysis of 59 DNA fragments obtained from the experiments using semi-specific PCR (Figure 2). The genetic distance coefficients between the tested lines were computed from the experiments using semi-specific PCR and ranged from 0.19 to 0.58 (average 0.40).

## 4. Discussion

Similar morphological features of the gerbera cultivars do permit to identify the varieties as the features are too similar between the varieties. Thus, methods based on molecular biology are being more frequently applied, for example, AFLP [10–12], SSR [13], RAPD [4, 14], and semi-specific PCR [8]. Methods such as AFLP and SSR require expensive equipment and reagents [10–13]. The here employed RAPD and semi-specific PCR methods presented in this study are relatively cheaper than the aforementioned alternatives, which may predispose them to being applied in cultivar identification, especially considering the cost and feasibility of analysis [8].

 The conducted analyses of RAPD with the use of DNA from eight Polish cultivars of gerbera (*Amelia, Bartoszyce, Bolesławiec, Delfin, Kraków, Kreta, Safona, Samuraj*) stood out as generating few amplification products. In the present work 70 per cent of the used primers generated bands. Polymorphic bands were generated by 60 percent of the used-in-the-tests primers. Rezende et al. [4] carried out research on *G*. *jamesonii* testing 31 primers, of which 21 generated polymorphic bands (68%). Similar studies were conducted on gerbera by Chung et al. [7], and the elicited results showed that merely 36 of the 80 primers (45%) would generate polymorphic bands.

 Although during the tests of genetic differentiation of gerbera cultivars different RAPD primers generated polymorphic bands, the use of a single primers to identify one lineage proved insufficient, as differentiating bands would show up for several cultivars simultaneously, and to distinguish them other primers had to be used. A similar result was yielded by Rafalski [8] when testing corn lineages.

 The method utilizing semi-specific PCR has never been used in research on *Gerbera* sp. The use of partially specific primers is a relatively new method of investigating the DNA polymorphism. The suggested system is equally simple and quick as the RAPD method. Simultaneously the semi-specific primers generate much more complex images of the amplification profiles in comparison to the RAPD method [8, 15].

 When using the semi-specific PCR with the material from the eight *gerbera* cultivars (*Amelia, Bartoszyce, Bolesławiec, Delfin, Kraków, Kreta, Safona, Samuraj*) (Table 2). a polymorphism reaching 100% was observed, which means that one primer sufficed to distinguish all eight cultivars. The highest number of bands was generated by the 18-nucleotide primers of the ET group, similar results were yielded by Rafalski [8] who applied 15- and 18-nucoeotide primers of the ET group. Most of the used in the present work primers would generate 20 to 30 DNA fragments.

The yielded results served to compute the coefficients of genetic distance between the cultivars. Diagram of Euclidean distances is shown in Figures 1 and 2. The coefficients of genetic distance of the tested gerbera lines computed from the results obtained by means of semi-specific primers of the ET group ranged from 0.19 to 0.58, although the coefficients of genetic distance between the lines computed from experiments conducted with the use of the RAPD technique ranged from 0.09 to 0.62. Also in the Rafalski tests carried out on the corn lines [8], higher method sensitivity was achieved by means of semi-specific PCR in comparison to the RAPD technique. In the research on corn, the genetic distance computed from RAPD experiments was 0.19 on average, and 0.38 from multiplications by means of the semi-specific primers. The authors of this paper favor the thesis propounded by Rafalski, in which the PCR system using the semiconservative primers enable a precise measurement of the genetic distance between the lines manifesting significant consanguinity.

## Figures and Tables

**Table 1:** Primers used to evaluate the consanguinity of the *G. jamesonii* cultivars with the nucleotide sequence, total bands number, number of differentiating bands, and the contribution of differentiating bands expressed as a percentage.

Primer code	Nucleotide sequence of the primers	Total bands number	Contribution of differentiating bands (%)
A2	5′-TGCCGAGCTG-3′	5	40.0%
C11	5′-AAAGCTGCGG-3′	10	20.0%
D5	5′-TGAGCGGACA-3′	7	28.6%
D8	5′-GTGTGCCCCA-3′	8	25.0%
D11	5′-AGCGCCATTG-3′	7	28.6%
G12	5′-CAGCTCACGC-3′	9	33.3%

**Table 2:** Primers used to evaluate the consanguinity among the cultivars of *G. jamesonii* with the nucleotide sequence, total bands number and the fraction of polymorphic bands in percentage.

Primer code	Total bands number	Participation of differentiating polymorphic bands (%)
ET 1/18	6	66.6%
ET 2/18	11	90.9%
ET 3/18	4	25%
ET 4/18	4	75%
ET 5/18	9	33.3%
ET 6/18	18	100%
ET 31/15	0	0
ET 32/15	10	80%
ET 33/15	3	0
ET 34/15	6	50%
ET 35/15	9	55.5%
ET 36/15	8	62.5%
